# Occurrence of Regulated Mycotoxins and Other Microbial Metabolites in Dried Cassava Products from Nigeria

**DOI:** 10.3390/toxins9070207

**Published:** 2017-06-29

**Authors:** Adebayo B. Abass, Wasiu Awoyale, Michael Sulyok, Emmanuel O. Alamu

**Affiliations:** 1 International Institute of Tropical Agriculture, PMB 5320 Oyo Road, Ibadan 200285, Oyo State, Nigeria; a.abass@cgiar.org (A.B.A.); o.alamu@cgiar.org (E.O.A.); 2 Department of Food Science and Technology, Kwara State University Malete, PMB 1530, Ilorin 240001, Kwara State, Nigeria; 3 Department of Agrobiotechnology (IFA-Tulln), University of Natural Resources and Life Sciences, Vienna (BOKU), Konrad Lorenzstr. 20, A-3430 Tulln, Austria; michael.sulyok@boku.ac.at

**Keywords:** cassava products, Nigeria, emerging mycotoxins, regulated mycotoxins, microbial metabolite, LC/MS, human exposure, food safety, food standards

## Abstract

Dried cassava products are perceived as one of the potential sources of mycotoxin ingestion in human foods. Processing either contributes to the reduction of toxins or further exposes products to contamination by microorganisms that release metabolic toxins into the products. Thus, the prevalence of microbial metabolites in 373 processed cassava products was investigated in Nigeria. With the use of liquid chromatography tandem-mass spectrometry (LC-MS/MS) for the constituent analysis, a few major mycotoxins (aflatoxin B_1_ and G_1_, fumonisin B_1_ and B_2_, and zearalenone) regulated in food crops by the Commission of the European Union were found at concentrations which are toxicologically acceptable in many other crops. Some bioactive compounds were detected at low concentrations in the cassava products. Therefore, the exposure of cassava consumers in Nigeria to regulated mycotoxins was estimated to be minimal. The results provide useful information regarding the probable safety of cassava products in Nigeria.

## 1. Introduction

The cassava root (*Manihot esculenta Crantz*) significantly contributes to food security, incomes, and employment opportunities in the rural areas of Sub-Saharan Africa [1], especially in Nigeria, the world’s largest cassava producer [2]. Significant post-harvest deterioration of fresh cassava roots occurs because of the natural high moisture content, which accelerates microbial deterioration and undesirable biochemical changes in the products [3]. Processing is used to extend the shelf life, facilitate transport and, most importantly, detoxify the roots by removing the inherent cyanogens [4,5,6]. Hence, cassava root is processed in Nigeria into gari, tapioca, lafun, fufu, starch, and high-quality cassava flour (HQCF), among others, with all the products having different physical properties due to variations in processing methods [7,8,9]. However, these processing methods, as well as the environments and natural microflora, influence the types and concentrations of microbial metabolites in the final food products [10,11].

The various processing methods for cassava in Nigeria often result in a range of food and feed products. Cassava starch and high-quality cassava flour (HQCF) are dried, unfermented products that must be dried immediately to avoid fermentation [12,13]. Starch is produced by peeling the roots, washing, grating, pulverizing, wet-sieving, sedimentation, decanting, dewatering, drying, and milling. HQCF processing is similar, except that the grated cassava is dewatered and dried immediately. The production of lafun may or may not involve peeling of the cassava roots before washing, fermenting in water (either in a flowing stream or stationary water) for softening, bagging/dewatering, drying, and milling [14]. The production of fufu flour is similar, except that, after fermentation, the mash is wet-sieved before sedimentation, dewatering, and final drying. Lafun and fufu flours are categorized as dried fermented flours, while tapioca is an unfermented product produced by toasting the extracted wet starch [15]. The toasting of fermented cassava mash to make gari is similar to this process, and similar utensils are used. Additionally, toasted fermented products, yellow or fine white gari, and yellow or white kpo-kpo gari are produced by peeling the roots, washing, grating, bagging, fermenting, dewatering, granulating, sieving, roasting, and again sieving to achieve a specific particle sizes. Fine gari has particle sizes of ≤500 µm while particles of kpo-kpo gari are >1 mm. The addition of palm oil to the white granules during toasting imparts a yellow color, thus the name yellow gari [16,17].

Mycotoxins are secondary fungal metabolites that may develop in almost any food or feedstuff during the growing season, at harvest time, or during processing or storage, depending on the environment and method of handling. Ingestion of high concentrations of mycotoxins can cause sickness or death in humans and animals [18]. There are three major genera of fungi that produce mycotoxins: *Aspergillus*, *Fusarium*, and *Penicillium* [19]. Kaaya and Eboku [20] reported that aflatoxins are naturally-occurring mycotoxins produced as secondary metabolites by many species of *Aspergillus* spp. (*Aspergillus flavus*, *A. fumigatus*, *A. parasiticus*, and *A. niger*). These secondary metabolites include aflatoxins B_1_, B_2_, G_1_, and G_2_ [21]. Cool, wet weather favors *Fusarium* toxins, while hot, humid weather encourages aflatoxin formation [22]. Other forms of metabolites can be produced by microorganisms occurring by chance in feed and foodstuff during handling, processing, and storage. Knowledge of the levels of contaminants in food products is needed to assist food regulatory agencies in estimating possible exposure of consumers to such contaminants and in setting maximum allowable levels for food control purposes. It should be noted that aflatoxin*s* are genotoxic carcinogens. Therefore, the maximum limits for total aflatoxin content in a food or feed product (the sum of aflatoxins B_1_ and G_1_) is controlled or regulated, depending on the form in which the product is consumed or further processed before consumption. Additionally, a separate limit is often set for aflatoxin B_1_ content since this is the most toxic of the compounds.

Globally, well-known or regulated microbial mycotoxins are frequently analyzed in food and feedstuff, and the maximum limits are enforced to ensure the safety of consumers [23]. These are different from emerging mycotoxins which are not routinely determined, no maximum limits have been established for them, partly because the knowledge of their incidence in foods is still emerging, and their safety or potential toxicity has not been fully elucidated [24,25,26]. Hence, it is difficult to conduct a proper assessment of the risk of exposure of humans and animals to high concentrations of emerging mycotoxins of unknown toxicity, which could occur sporadically in food and feedstuff [25].

Few studies have been conducted on the contamination of cassava products with regulated mycotoxins [26,27,28,29,30,31] when compared with the number of studies of toxin contamination of cereals, peanuts, dairy products, wheat, and dried chilies [32,33], and studies of other microbial metabolites are few, as well. Moreover, far less has been discussed in the literature about emerging mycotoxins in cassava products from Africa. For instance, Juan et al. [24] reported that Ediage et al. [30] detected and quantified aflatoxin B_1_ (9 µg/kg), aflatoxin B_2_ (8 μg/kg), fumonisin B_1_ (4–21 μg/kg), diacetoxyscirpenol (6 μg/kg), and zearalenone (12 μg/kg) in cassava flour samples from the Republic of Benin. On the other hand, a larger range of data has been published on the occurrence in cereals and cereal products of emerging mycotoxins, such as enniatins, beauvericin, moniliformin, fusaproliferin, fusaric acid, culmorin, butenolide, sterigmatocystin, emodin, mycophenolic acid, alternariol, alternariol monomethyl ether, and tenuazonic acid [25].

The paucity of reliable data may have contributed to the inability of the major cassava-producing countries in Africa, including Nigeria, the world’s largest producer and consumer of cassava products, to establish regulatory limits for mycotoxins in cassava products calculated based on per capita consumption of the cassava products, and the prevalence and concentrations of the different mycotoxins in the products. On the other hand, microbial specifications and permissible limits for food additives, pesticide residues, and heavy metal contaminants have all been stipulated [34]. Therefore, the objective of this study was to evaluate the prevalence of major mycotoxins and other microbial metabolites in various dried cassava products consumed in Nigeria, using a more versatile and precise mycotoxin quantitation methodology based on the proven principle of isotope dilution mass spectrometry, as previously described [23,24,25,30,31,35].

## 2. Results and Discussion

### 2.1. Mycotoxins and Microbial Metabolites in Dried Cassava Products

#### 2.1.1. Regulated Mycotoxins

Six hundred and forty-six analytes were screened with a QTrap 5500 LC-MS/MS system to target microbial metabolites in the 373 cassava samples. Only 91 microbial metabolites were detected in more than one sample (See Supplemental Table S1). As for regulated mycotoxins, only aflatoxins B_1_ and G_1_ were found, and these in a total of four samples: fufu flour (3/36 samples) and HQCF (1/29 samples), respectively. Fumonisin B_1_ was found in lafun (88.1 µg/kg) and fufu flour (102.7 µg/kg) (1/30 and 1/36 samples, respectively), at an average concentration of 95.4 µg/kg sample. Fumonisin B_2_ was present in lafun (2/30 samples), fufu flour (2/36 samples), and fine white gari (1/113 samples) at an average concentration of 50.0 µg/kg. Zearalenone was found in HQCF (2/29 samples), lafun (6/30 samples), fufu flour (2/36 samples), fine yellow gari (1/50 samples), and white kpo-kpo gari (3/52 samples) (see Table 1). 

About 70% of cassava roots produced in Nigeria are processed into gari, making this the most popular cassava product in Nigeria [36]. The aflatoxin content of all types of gari samples was under the detectable limit; these results, therefore, suggest that gari is very safe from aflatoxin contamination. With averages of 1.2 µg/kg of aflatoxin B_1_ (in fufu flour) and 2.9 µg/kg of aflatoxin G_1_ (in HQCF), the aflatoxin levels of the dried cassava products sampled and tested were below the European Union values of 5 µg/kg tolerance level in foods [37]. The level of aflatoxin B_1_ found in the HQCF of the present study was lower compared to the values (4–21 μg/kg) reported by Ediage et al. [30] for cassava flour from the Republic of Benin.

Neither aflatoxins B_2_, G_2_, M_1_, M_2_, P_1_, nor ochratoxin A was detected in any of the 373 dried cassava product samples. Fumonisin B_3_ was detected in only one fufu flour sample (14.5 µg/kg). However, Ediage et al. [30] oberved that 8 μg/kg of aflatoxin B_2_ was present in cassava flour from the Republic of Benin. Similarly, patulin and deoxynivalenol were absent in all the samples, and the range of zearalenone concentrations (0.9–90.4 µg/kg) obtained was lower than that reported by Sulyok et al. [31] for cassava samples from Rwanda (2830 µg/kg) and Tanzania (8490 µg/kg), and the values (12 μg/kg) reported by Ediage et al. [30] for cassava flour from the Republic of Benin. The results suggest that processed cassava products in Nigeria are safe with respect to the regulated mycotoxins, also considering that the regulated levels of zearalenone and fumonisins reported for maize and other cereals in African countries, such as Niger, Ghana, the United Republic of Tanzania, Uganda, and Benin, range between 50 µg/kg and 1000 µg/kg, and 1000 µg/kg to 3000 µg/kg, respectively [37]. Implicitly, a better understanding of the impact of processing practices adopted in Rwanda and Tanzania on the relatively higher levels of zearalenone and fumonisin in samples from the two countries may be helpful in efforts towards setting future regulatory levels for these mycotoxins in cassava products. 

#### 2.1.2. Other Microbial Metabolites

**Figure 1:**
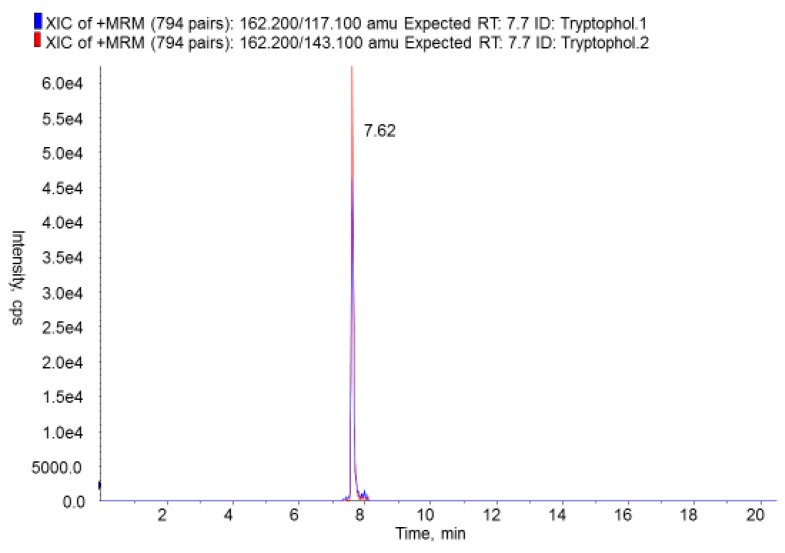
Overlay of XICs on tryptophol.

As regards the prevalence of non-regulated microbial metabolites in the cassava samples, only 33 analytes were detected at concentrations higher than their respective limits of detection (LODs) in 5% or more of the 373 samples investigated (see Table 2). Of these 33, only 16 were found in 15% or more of the samples investigated. These were asperphenamate (99.5%), asperglaucide (99.2%), cyclo (L-Pro-L-Val) (92.0%), tryptophol (88.5%), cyclo (L-Pro-L-Tyr) (85.5%), brevianamid F (79.6%), kojic acid (71.3%), N-benzoyl-phenylalanine (55.2%), fellutanine A (48.3%), emodin (41.8%), rugulusovin (37.5%), alternariol methyl ether (25.7%), ilicicolin B (24.1%), ilicicolin A (18.2%), ilicicolin C (16. 9%), and ascochlorin (15.6%) (see Table 2). Figure 1 shows the overlay of XICs on tryptophol. 

These 16 metabolites could be the most common in dried cassava products in Nigeria. Of these, the most common metabolites associated with *Aspergillus* spp. were kojic acid, asperphenamate, N-benzoyl-phenylalanine, emodin, and asperglaucide. The predominant metabolites of *Alternaria* spp. were cyclo (L-Pro-L-Tyr), cyclo (L-Pro-L-Val), and alternariol methyl ether. Furthermore, tryptophol was the most common metabolite associated with *Fusarium* spp. and brevianamid F, fellutanine A, and rugulusovi , associated with *Pennicillum* spp., were the most commonly identified metabolites of that species (see Table 2). These results agree with those obtained in previous studies [11,38,39,40] of various staple foods from some countries, including cassava products.

Kojic acid, asperphenanate, N-benzoyl-phenylalanine, emodin, and asperglaucide are all metabolites associated with *Aspergillus* spp. (see Table 3). The concentrations of kojic acid, a 5-hydroxy-2-hydroxymethyl-4-pyranone, in the dried product samples ranged from 8.35 to 1754.80 µg/kg; lafun samples had the highest, and cassava starch the lowest concentration. The kojic acid content of lafun in this study was lower than the maximum values (650,000 µg/kg and 93,700 µg/kg) recorded in cassava samples from Tanzania and Rwanda, respectively [31]. However, the processing methods for these cassava products were not indicated. Kojic acid can also be produced from various carbohydrate sources in an aerobic condition by a variety of microorganisms [41]. The fermentation process to produce lafun may be more favorable for the production of kojic acid [42] than that used for gari or fufu. Poisoning from the consumption of oriental fermented foods containing kojic acid, where its presence is common, has not been reported in humans, although there are still inconsistent and controversial results on kojic acid toxicity [43]. Additionally, Nohynek et al. [44] reported that the existing literature on the toxicity of kojic acid is somewhat inconclusive, even though it has been stated from the genotoxicity and human health risk of topical use of kojic acid that consumer exposure to fermented foods does not pose a significant risk to human health.

**Figure 2:**
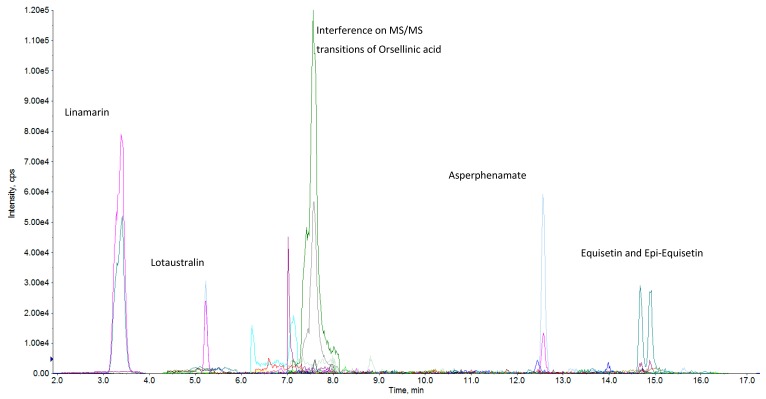
Overlaid ESI (-) MRM-chromatogram (sum of all XICs) in a representative sample.

Unlike kojic acid, asperphenamate is an unusual ester of N-benzoyl-phenylalanine and N-benzoyl-phenylalaninol produced by *Aspergillus* spp., *Penicillium* spp., and plants [45,46]. The concentration of this metabolite was highest in yellow kpo-kpo gari (270.2 µg/kg), and lowest in fine yellow gari (6.8 µg/kg). Similarly, the concentration of N-benzoyl-phenylalanine, which has the same biogenetic pathway as asperphenamate, was also highest in yellow kpo-kpo gari (141.1 µg/kg) and lowest in fine yellow gari (1.0 µg/kg). Figure 2 shows the overlaid ESI (-) MRM-chromatogram (sum of all XICs) of asperphenamaten, equisetin and epi-equisetin in a representative sample, which also contained natural toxins in cassava (linamarin and lotaustralin).

Emodin (1,3,8-trihydroxy-6-methylanthracene-9,10-dion, a natural compound belonging to the anthraquinone family, was prevalent (41.8%) in the dried cassava product samples. It occurs naturally either in a free state or combined with sugar in a glucoside and in rhubarb, cascara sagrada, aloe, and other plants. It has been found to have many health benefits, including antitumor effects on human cells [47]. Thus, emodin content in foods may not necessarily be of fungal origin [48]. The emodin concentrations in the dried product samples (quantified in free form) ranged from 0.17 to 31.17 µg/kg, with fufu flour having the highest, and cassava starch the lowest, concentrations. 

The asperglaucide content of the samples was highest in lafun (385.8 µg/kg) and lowest in fine white gari (25.4 µg/kg). Asperglaucide is reported to have an anti-inflammatory effect and the ability to inhibit cysteine peptidase.

Table 4 reveals the prevalence and concentrations of *Alternaria, Fusarium*, and *Penicillium* spp. metabolites in samples of various types of cassava products from Nigeria. *Cyclo (L-Pro-L-Tyr)*, or maculosin, is a diketopiperazine formed by the fusion of tyrosine and proline that has been reported as a secondary metabolite of various fungi and bacteria on knapweed as reported by Stierle et al. [49]. These researchers also identified *Cyclo (L-Pro-L-Tyr)* as a host-specific phytotoxin produced by *Alternaria alternata* on spotted knapweed [49]. In the samples, the concentrations of this metabolite ranged from 22.4 µg/kg to 199.9 µg/kg; fufu flour exhibited the lowest and yellow kpo-kpo gari the highest concentration. Related to *cyclo (L-Pro-L-Tyr)* is another diketopiperazine known as *cyclo (L-Pro-L-Val)*, which is formed by the fusion of valine and proline [50]. This was found in higher concentrations in fine white gari (625.3 µg/kg) than in yellow kpo-kpo gari (57.2 µg/kg). Alternariol monomethyl ether, which is produced by different species of *Alternaria* spp., has been reported to have low acute toxicity [51,52]. This metabolite has frequently been detected in apples and their products, apple juice concentrates, mandarins, olives, pepper, tomatoes and their products, oilseed rape meal, sunflower seeds, sorghum, wheat [53], and in edible oils (olive oil, rapeseed oil, sesame oil, sunflower oil), among others [54]. The alternariol methyl ether content of the dried cassava product samples ranged from 0.02 µg/kg to 1.49 µg/kg. Fufu flour had the lowest content, and fine yellow gari, the highest. Samples of cassava starch and tapioca did not contain alternariol methyl ether at detectable levels.

The only *Fusarium* spp. metabolite which was present in more than 75% of the cassava product samples was tryptophol. This is an aromatic alcohol that induces sleep in humans and is produced by many microbial species [55]. It is also produced by the trypanosoma parasite in wine as a secondary product of alcoholic fermentation [55]. Tryptophol may also be formed from an amino acid (tryptophan) during fermentation [31]. Lafun had the highest (1121.9 µg/kg), and fine yellow gari the lowest (121.3 µg/kg), tryptophol content (see Table 4).

Brevianamid F, fellutanine A, and rugulusovin metabolites associated with *Penicillium* spp. were prevalent (>75%) in the cassava product samples (see Table 4). Brevianamid F is a cyclic dipeptide produced by many species of *Penicillium* and an intermediate in the production of many other fungal metabolites [31]. The brevianamid F content of the dried cassava product samples was highest in fine white gari (44.0 µg/kg) and the lowest in fufu flour (7.1 µg/kg). Fellutanine A is one of the bio-active diketopiperazine alkaloids often produced by *Penicillium fellutanum* and *Penicillium simplicissimum* [56], which is also a non-annulated analogue of cyclo (L-Trp-L-Trp). This implied that fellutanine can also be produced from the amino acid tryptophan during fermentation [56]. The concentration range of this metabolite in the samples was 0.02 µg/kg to 4.14 µg/kg, with fine white gari having the highest, and tapioca the lowest, concentrations. The rugulusovin content of the dried product samples ranged between 0.06 µg/kg and 2.05 µg/kg. The values were highest in tapioca and lowest in fine yellow gari. Rugulusovin was not detected in high-quality cassava flour, possibly because of the absence of fermentation in the processing method (Table 2 and Table 4).

As shown in Table 2, Table 3 and Table 4, some emerging mycotoxins, namely, beauvericin, moniliformin, emodin, alternariol methyl ether, and tenuazonic acid occurred in more than 5% of the total number of cassava products studied. Sterigmatocystin and O-Methylsterigmatocystin occurred in more than one sample, but less than 5% of the samples (see Supplemental Table S1). While there are no significant differences in the concentration of Emodin among the cassava products, Yellow kpo-kpo gari had significantly higher (*p* < 0.05) concentrations of alternariol methyl ether than any of the other cassava products, suggesting possible role of processing method or presence of carotenoids (antioxidants that are present in yellow cassava roots), in the formation of alternariol methyl ether. In addition, the fermented cassava products (Lafun, fufu flour, fine yellow gari, fine white gari and yellow kpo-kpo gari) exhibited consistent, significantly higher(*p* < 0.05) concentrations of microbial metabolites than non-fermented products (cassava starch, HQCF, and tapioca) (see Table 3 and Table 4). Lafun contained significantly higher (*p* < 0.05) concentrations of kojic acid (1755 ± 7196 µg/kg) than all the other products. Yellow kpo-kpo gari contained significantly higher (*p* < 0.05) concentrations of asperphenamate (270± 655 µg/kg), N-benzoyl-phenylalanine (141± 384 µg/kg), cyclo (L-Pro-L-Tyr) (200 ± 18 µg/kg) and cyclo (L-Pro-L-Val) (57± 56 µg/kg) than all the other products, while fine white gari contained significantly higher (*p* < 0.05) concentration of brevianamid F (44 ± 52 µg/kg). Available knowledge suggests that food processing causes the masking of some mycotoxins through oxidation, reduction, or conjugation phenomenon [24]. From the preceding, there is an indication that the existing diverse traditional cassava processing practices in different countries, most of which involve fermentation by different chance-microorganisms, could alter the metabolites found in cassava products. In the light of this, further understanding of the diversity and concentration of emerging mycotoxins in cassava products would be required with regard to the effect of different processing practices and the presence of beta carotenoids in some cassava varieties.

## 3. Conclusions

The results of this study showed that regulated mycotoxins, based on European regulations, were not prevalent in any dried cassava product sample from Nigeria. The results, therefore, indicate that consumers of dried cassava products made in Nigeria are not exposed to high levels of regulated mycotoxins. Nevertheless, the study recommends further studies on the role of different processing practices in the alteration of the contents of emerging mycotoxins in cassava products. Additionally, precautions in the form of establishing hygiene and industrial standards for raw materials combined with other operational protocols in cassava processing companies are needed to prevent accidental exposures of consumers to high concentrations of toxins in improperly processed products. Establishing protocols for manufacturing practices will be in line with the global practice of establishing permissible limits, preventing food toxins, and reviewing the limits and practices from time-to-time, taking account of new advances in scientific and technical knowledge on the toxins and any new variants of the associated microorganisms.

## 4. Materials and Methodology

### 4.1. Sampling of Dried Cassava Products Traded in Nigeria

Three hundred and seventy-three samples of dried cassava products were taken from processors and vendors located in the humid forest (92), derived savannah (267), and Southern Guinea Savannah (14) zones. The distribution was as follows: tapioca: 36 samples, white kpokpo gari: 52, yellow kpokpo gari: 12, fine yellow gari: 50, fine white gari: 113, fufu flour: 36, lafun: 30, starch: 15, and high-quality cassava flour (HQCF) 29. All of the products were properly sampled by quartering before sending to the laboratory for analyses. Each cassava product (200 g) collected was a representative of the sampling frame, which was based on the relative quantities of the products processed from fresh cassava and traded in each agroecological zone. All of the samples were collected during the rainy season. Samples were kept in polypropylene bags and transported to the Center for Analytical Chemistry Laboratory in the Department of Agrobiotechnology, University of Natural Resources and Life Sciences, Vienna, for analysis.

### 4.2. Determination of Mycotoxins and Other Microbial Metabolites

The mycotoxin metabolites were determined using the method reported in our previous study by Malachova et al. [57]. The accuracy of the method was verified on a routine basis by inter-laboratory comparison trials organized by BIPEA (Gennevilliers, France). *Z*-scores are in the acceptable range of −2 < *z* < 2 for ca. 90% of the submitted results, with other results, mainly deriving from matrices, that have not been previously validated [58].

The samples (5 g) were weighed into a 50-mL polypropylene tube (Sarstedt, Nümbrecht, Germany), and the extraction solvent (acetonitrile/water/acetic acid 79:20:1, *v*/*v*/*v*) was added at a ratio of 5 mL per gram of sample. Samples were extracted for 90 min on a GFL 3017 rotary shaker (GFL, Burgwedel, Germany) and diluted with the same volume of dilution solvent (acetonitrile/water/acetic acid 79:20:1, *v*/*v*/*v*), and the diluted extracts injected [58]. Centrifugation was not necessary due to sufficient sedimentation by gravity. Apparent recoveries of the analytes were taken from the analysis of 15 individual spiked samples.

A QTrap 5500 LC-MS/MS System (Applied Biosystems, Foster City, CA, USA) equipped with a TurboIonSpray^®^ electrospray ionization (ESI) source and a 1290 Series HPLC System (Agilent, Waldbronn, Germany) was used for the liquid chromatography-mass spectrometry/mass spectrometry (LC-MS/MS) screening of target microbial metabolites [59]. Chromatographic separation was performed at 25 °C on a Gemini^®^ C18 column (Phenomenex, Torrance, CA, USA ), of 150 × 4.6 mm ID, with 5 μm particle size, equipped with a C18 4 × 3 mm ID SecurityGuard™ cartridge (Phenomenex, Torrance, CA, USA). ESI-MS/MS was performed in the time-scheduled multiple reaction monitoring (MRM) mode, both in positive and negative polarities, in two separate chromatographic runs per sample by scanning two fragmentation reactions per analyte. The MRM detection window of each analyte was set to its expected retention times of ±27 and ±48 s in the positive and the negative modes, respectively. Confirmation of positive analyte identification was obtained by conducting two MRM assays per analyte (except in the case of moniliformin, which exhibited only one fragment ion). This yielded 4.0 identification points according to European Union Commission Decision 2002/657 [60]. Additionally, the LC retention time and the intensity ratio of the two MRM transitions agreed with the related values of an authentic standard within 0.1 min and 30% rel., respectively. The MRM transitions for all the major toxins and the metabolites identified in this work have previously been detailed in Malachova et al. [53]. Quantitation was performed using external calibration in connection with apparent recoveries previously determined for cassava [28]. Isolation and identification of microorganisms were not included in this study.

### 4.3. Statistical Analysis

Analysis of variance (ANOVA) and separation of the mean values (using Duncan’s multiple range test at *p* < 0.05) were done using Statistical Package for the Social Sciences (SPSS) software, version 21.0 (SPSS, Inc., Chicago, IL, USA).

## Figures and Tables

**Table 1:** Overview of occurrence and concentrations of regulated mycotoxins detected in processed cassava samples from Nigeria.

Products	N	Aflatoxins			Other Mycotoxins				
		Aflatoxin B_1_ (µg/kg)	Aflatoxin G_1_ (µg/kg)	Prevalence (%)	Fumonisin B_1_ (µg/kg)	Fumonisin B_2_ (µg/kg)	Fumonisin B_3_ (µg/kg)	Zearalenone (µg/kg)	Prevalence (%)
R (%)		82.90	80.50		86.30	92.20	93.40	101.70	
LOD (µg/kg)		0.20	0.20		3.00	1.50	2.00	0.30	
Cassava starch	15	+	+	0.00	+	+	+	+	0.00
HQCF	29	+	2.94 (1)	3.45	+	+	+	1.10 (2)	6.90
Lafun	30	+	+	0.00	88.09 (1)	10.70 (2)	+	7.60 (6)	30.00
Fufu flour	36	1.16 (3)	+	8.33	102.71 (1)	21.28 (2)	14.49 (1)	1.89(2)	16.67
Tapioca	36	+	+	0.00	+	+	+	+	2.78
Fine yellow gari	50	+	+	0.00	+	+	+	90.40 (1)	2.78
Fine white gari	113	+	+	0.00	+	218.12(1)	+	0.92 (2)	2.65
Yellow kpo-kpo gari	12	+	+	0.00	+	+	+	+	0.00
White kpo-kpo gari	52	+	+	0.00	+	+	+	11.01(3)	5.77
Range (all products)	373	0.00–1.16 (3)	0.00–2.94 (1)	3.45–8.33	88.33–02.71 (2)	10.70–18.12 (5)	0.00–14.49 (1)	0.92–90.40 (16)	0.00–30.00

Calculation of means was based on positive samples. R: apparent recovery; LOD: limit of detection; +: represents a positive analyte but that was detected at a concentration < LOD. Figures in parentheses are a number of samples in which an analyte was detected at > LOD.

**Table 2:** Number of non-regulated microbial metabolites detected at or above limit of detection in at least 5% of the total number of processed cassava samples.

Serial Number	Analyte	P/N	Prevalence (%)	LOD (µg/kg)	R (%)	Serial Number	Analyte	P/N	Prevalence (%)	LOD (µg/kg)	R (%)
1	Averufin	27/373	7.24	0.06	71.2	18	Asperphenamate	371/373	99.46	0.04	100
2	3-Nitropropionic acid	52/373	13.94	1.00	36	19	Brevianamid F	297/373	79.62	0.50	95.8
3	Kojic acid	266/373	71.31	15.00	100	20	Citreorosein	26/373	6.97	0.60	100
4	Quinolactacin A	27/373	7.24	0.08	100	21	Tryptophol	330/373	88.47	15.00	96.7
5	Quinocitrinine A	22/373	5.90	0.15	100	22	Rugulusovin	140/373	37.53	0.40	100
6	Beauvericin	20/373	5.36	0.002	97.6	23	Cyclo (L-Pro-L-Tyr)	319/373	85.52	1.50	100
7	Epiequisetin	45/373	12.06	0.20	136	24	Cyclo (L-Pro-L-Val)	343/373	91.96	0.50	100
8	Equisetin	39/373	10.46	0.20	136	25	N-benzoyl-phenylalanine	206/373	55.23	0.80	100
9	Moniliformin	30/373	8.04	0.40	82.4	26	Emodin ^†^	156/373	41.82	0.20	105.8
10	LL-Z 1272e	32/373	8.58	0.06	100	27	Isorhodoptilometrin	22/373	5.90	0.06	100
11	Alternariol methyl ether	96/373	25.74	0.02	97.9	28	Skyrin	33/373	8.85	0.30	87
12	Ilicicolin A	68/373	18.23	0.15	100	29	Usnic acid	20/373	5.36	0.03	100
13	Ilicicolin B	90/373	24.13	0.30	100	30	Fellutanine A	180/373	48.26	0.60	100
14	Ilicicolin C	63/373	16.89	0.30	100	31	Neoechinulin A	49/373	13.14	0.60	100
15	Ascochlorin	58/373	15.55	0.30	100	32	Unugisin E	25/373	6.70	1.20	1000
16	Chloramphenicol	39/373	10.46	0.03	92	33	Neoechinulin A	32/373	8.58	0.40	100
17	Asperglaucide	370/373	99.20	0.40	100						

P: positive samples; N: total number of samples; R: apparent recovery; LOD: limit of detection; ^†^: emodin was provided in free form.

**Table 3:** Prevalence and concentrations of metabolites linked to *Aspergillus* spp. in different groups of processed cassava samples in Nigeria.

Product	N	Kojic Acid (µg/kg)	Asperphenamate (µg/kg)	N-Benzoyl-Phenylalanine (µg/kg)	Emodin (µg/kg)	Asperglaucide (µg/kg)
LOD (µg/kg)		15.00	0.04	0.80	0.19	0.40
Cassava starch	15	8.35 ± 23.75 b	39.07 ± 92.51 b	10.21 ± 25.14 b	0.17 ± 0.26 a	41.77 ± 65.73 b
HQCF	29	632.68 ± 1616.54 b	27.08 ± 50.74 b	6.45 ± 13.20 b	0.34 ± 0.83 a	119.87 ± 298.89 b
Lafun	30	1754.80 ± 7196.41 a	71.90 ± 189.70 b	12.66 ± 30.58 b	0.30 ± 0.42 a	385.83 ± 1117.12 a
Fufu flour	36	32.61 ± 46.85 b	63.98 ± 236.38 b	8.60 ± 30.24 b	31.17 ± 185.98 a	52.72 ± 138.25 b
Tapioca	36	13.95 ± 30.50 b	34.93 ± 80.19 b	3.92 ± 6.00 b	0.19 ± 0.53 a	100.81 ± 254.94 b
Fine yellow gari	50	183.39 ± 184.70 b	6.75 ± 8.36 b	0.99 ± 1.11 b	17.72 ± 114.32 a	59.17 ± 194.50 b
Fine white gari	113	167.49 ± 102.65 b	9.51 ± 18.18.52 b	1.55 ± 5.71 b	1.57 ± 14.35 a	25.40 ± 40.21 b
Yellow kpo-kpo gari	12	59.67 ± 82.69 b	270.19 ± 654.82 a	141.05 ± 384.32 a	2.50 ± 4.43 a	358.68 ± 793.03 a
White kpo-kpo gari	52	53.73 ± 58.68 b	13.36 ± 22.44 b	1.99 ± 3.73 b	1.44 ± 8.70 a	38.59 ± 39.59 b

LOD: limit of detection; N: the number of samples; Means with different letters in the same column are significantly different (*p* < 0.05).

**Table 4:** Prevalence and concentrations of metabolites linked to *Alternaria*, *Fusarium,* and *Penicillium* spp. in various groups of processed cassava samples in Nigeria.

Product	N	*Alternaria* spp.			*Fusarium* spp.	*Penicillium* spp.		
		Cyclo (L-Pro-L-Tyr) (µg/kg)	Cyclo (L-Pro-L-Val) (µg/kg)	Alternariol methyl ether (µg/kg)	Tryptophol (µg/kg)	Brevianamid F (µg/kg)	Fellutanine A (µg/kg)	Rugulusovin (µg/kg)
Cassava starch	15	27.28 ± 62.87 b	88.50 ± 241.32 b	+	202.30 ± 272.09 b	8.45 ± 20.37 b	+	0.93 ± 2.42 a
HQCF	29	43.46 ± 69.02 b	94.47 ± 206.88 b	0.10 ± 0.21 b	234.85 ± 578.44 b	11.49 ± 19.11 b	3.68 ± 5.61 a b	<LOD
Lafun	30	31.93 ± 38.65 b	85.81 ± 134.86 b	0.05 ± 0.17 b	1121.88 ± 2027.59 a	8.44 ± 11.13 b	0.99 ± 1.87c	0.72 ± 2.64 a
Fufu flour	36	22.43 ± 41.80 b	128.17 ± 480.03 b	0.02 ± 0.06 b	718.35 ± 1101.42 a b	7.09 ± 16.85 b	1.20 ± 2.60c	1.02 ± 3.74 a
Tapioca	36	30.84 ± 117.80 b	105.61 ± 341.60 b	+	264.65 ± 689.61 b	10.36 ± 37.98 b	0.02 ± 0.12c	2.05 ± 9.38 a
Fine yellow gari	50	56.76 ± 69.66 b	184.88 ± 292.37 b	1.49 ± 1.84 a	121.31 ± 166.12 b	18.61 ± 24.33 b	1.86 ± 3.33 bc	0.06 ± 0.40 a
Fine white gari	113	132.91 ± 146.49 b	625.33 ± 672.51 a	0.27 ± 1.07 b	543.93 ± 4.85 a b	43.95 ± 51.64 a	4.14 ± 5.12 a	0.18 ± 1.65 a
Yellow kpo-kpo gari	12	199.94 ± 18.48 a	57.18 ± 56.40 b	1.06 ± 1.21 a	980.97 ± 2234.56 a	7.35 ± 6.31 b	0.37 ± 0.90c	1.16 ± 2.18 a
White kpo-kpo gari	52	58.19 ± 61.28 b	294.73 ± 370.81 b	0.03 ± 0.10 b	614.19 ± 1268.11 a b	18.77 ± 21.51 b	1.81 ± 3.80 bc	1.06 ± 2.73 a

LOD: limit of detection; N: the number of samples; + represented positive analyte detected at a concentration < LOD. Means with different letters in the same column are significantly different (*p* < 0.05).

## Supplementary Materials

The following are available online at www.mdpi.com/2072-6651/9/7/207/s1, Table S1: List of all the 91 microbial metabolites detected in more than one cassava product from Nigeria.

## Practical Applications

Unhygienic conditions during production and storage of cassava food products can result in high levels of bacterial and fungal contamination of the products. This study has shown that the methods used for dried cassava production in Nigeria result in food products that are safe for human consumption. The information provided on the concentration of the most prevalent metabolites in cassava products could be used for establishing maximum tolerable levels for regulated contaminants in cassava products for local and export trade.
