# Supplementary material for: Occurrence of Regulated Mycotoxins and Other Microbial Metabolites in Dried Cassava Products from Nigeria
Source: Toxins (Basel). 2017 Jun 29;9(7):207. doi: 10.3390/toxins9070207 (PMC5535154; doi:10.3390/toxins9070207)
Supplement: Supplementary file 1 [file toxins-09-00207-s001.pdf]

Supplementary Materials: Occurrence of Regulated Mycotoxins and Other Microbial Metabolites in Dried Cassava Products from Nigeria

Adebayo B. Abass, Wasiu Awoyale, Michael Sulyok and Emmanuel O. Alamu

Table S1. List of all the 91 microbial metabolites detected in more than one cassava product from Nigeria.

Serial number	Mycotoxin Metabolites	Serial number	Mycotoxin Metabolites	Serial number	Mycotoxin Metabolites
1	Aflatoxin G_1	32	Fellutanine A	63	Epiequisetin
2	Averantin	33	Xanthotoxin	64	Equisetin
3	Averufanin	34	Aflatoxin B_1	65	Beauvericin
4	Versicolorin A	35	Fumonisin B_1	66	Alternariol methylether
5	Kojic acid	36	Fumonisin B_2	67	Ascochlorin
6	Fumiquinazolin D	37	Zearalenone	68	Ilicicolin A
7	Asperfuran	38	Sterigmatocystin	69	Ilicicolin B
8	Moniliformin	39	Averufin	70	Integracin A
9	Fusarin C	40	O-Methylsterigmatocystin	71	Integracin B
10	LL-Z 1272e	41	Versicolorin C	72	Chloramphenicol
11	Chrysogin	42	Norsolorinic acid	73	Nonactin
12	Tenuazonic acid	43	3-Nitropropionic acid	74	Monactin
13	Tentoxin	44	Cyclopiazonsäure	75	Dinactin
14	Macrosporin	45	Dihydrocitrinone	76	Brevianamid F
15	Andrastin A	46	Methylsulochrin	77	Rugulusovin
16	Dechlorogriseofulvin	47	Unugisin E	78	cyclo(L-Pro-L-Tyr)
17	Agroclavine	48	Tryptoquialanine Derivat	79	cyclo(L-Pro-L-Val)
18	Festuclavine	49	Tryptoquivaline A	80	N-Benzoyl-Phenylalanine
19	Questiomycin A	50	Deoxynortryptoquivalin	81	Emodin
20	Quinolactacin A	51	Deoxytryptoquivaline A	82	Citreorosein
21	Sclerotioramin	52	Prelaptin	83	Skyrin
22	Secalonic acid D	53	Quinadoline A	84	Physcion
23	Ilicicolin C	54	Quinadoline B	85	Iso-Rhodoptilometrin
24	Ilicicolin E	55	Nigragillin	86	Asperglaucide
25	Monocerin	56	Curvularin	87	Asperphenamate
26	Trichodermamide C	57	Oxaline	88	Cordycepin
27	Chlorocitreorosein	58	Purpactin A	89	Neoechinulin A
28	Chrysophanol	59	Purpuride	90	Tryptophol
29	Citreorosein	60	Berkedrimane B	91	Usnic acid
30	Dihydroxymellein	61	Quinocitrinine A		
31	Fallacinol	62	Enniatin B		
